# Clonidine for sedation in the critically ill: a systematic review and meta-analysis (protocol)

**DOI:** 10.1186/s13643-015-0139-7

**Published:** 2015-11-06

**Authors:** Gennie Jing Wang, Emilie Belley-Coté, Lisa Burry, Mark Duffett, Timothy Karachi, Dan Perri, Waleed Alhazzani, Frederick D’Aragon, Hannah Wunsch, Bram Rochwerg

**Affiliations:** Department of Medicine, Faculty of Health Sciences, McMaster University, Hamilton, Ontario Canada; Department of Clinical Epidemiology and Biostatistics, McMaster University, Hamilton, Ontario Canada; Department of Pharmacy, Mount Sinai Hospital, Toronto, Ontario Canada; Leslie Dan Faculty of Pharmacy, University of Toronto, Toronto, Ontario Canada; Department of Pediatrics, McMaster University, Hamilton, Ontario Canada; Hamilton Health Sciences, Hamilton, Ontario Canada; St. Joseph’s Healthcare Hamilton, Hamilton, Ontario Canada; Department of Critical Care Medicine, Sunnybrook Health Sciences Centre, Toronto, Ontario Canada; Department of Anesthesia, University of Toronto, Toronto, Ontario Canada; Interdepartmental Division of Critical Care Medicine, University of Toronto, Toronto, Ontario Canada

**Keywords:** Clonidine, Systematic review, Sedation, Delirium, Mechanical ventilation, Weaning

## Abstract

**Background:**

Management and choice of sedation is important during critical illness in order to reduce patient suffering and to facilitate the delivery of care. Unfortunately, medications traditionally used for sedation in the intensive care unit (ICU) such as benzodiazepines and propofol are associated with significant unwanted effects. Clonidine is an alpha-2 selective adrenergic agonist that may have a role in optimizing current sedation practices in the pediatric and adult critically ill populations by potentially minimizing exposure to other sedative agents.

**Methods/design:**

We will search MEDLINE, EMBASE, CINAHL, ACPJC, the Cochrane trial registry, World Health Organization International Clinical Trials Registry Platform (WHO ICTRP), and clinicaltrials.gov for eligible observational studies and randomized controlled trials investigating the use of clonidine as an adjunctive or stand-alone sedative agent in patients requiring invasive mechanical ventilation. Our primary outcome is the duration of mechanical ventilation. Secondary outcomes include the following, listed by priority: duration of sedation infusions, dose of sedation used, level of sedation, incidence of withdrawal from other sedatives, delirium incidence, ICU and hospital length of stay, use and duration of non-invasive ventilation, and all-cause ICU and hospital mortality. We will also capture unwanted effects potentially associated with clonidine administration such as clinically significant hypotension or bradycardia, clonidine withdrawal, self-extubation, and the accidental removal of central intravenous lines and arterial lines.

We will not apply any publication date, language, or journal restrictions. Two reviewers will independently screen and identify eligible studies using predefined eligibility criteria and then review full reports of all potentially relevant citations. A third reviewer will resolve disagreements if consensus cannot be achieved.

We will use Review Manager (RevMan) to pool effect estimates from included studies across outcomes. We will present the results as relative risk (RR) with 95 % confidence intervals (CI) for dichotomous outcomes and as mean difference (MD) or standardized mean difference (SMD) for continuous outcomes with 95 % CI. We will assess the quality of evidence using the Grading of Recommendations, Assessment, Development and Evaluation (GRADE) approach.

**Discussion:**

The aim of this systematic review is to summarize the evidence on the efficacy and safety of clonidine as a sedative agent in the critically ill population.

**Systematic review registration:**

PROSPERO CRD42015019365.

**Electronic supplementary material:**

The online version of this article (doi:10.1186/s13643-015-0139-7) contains supplementary material, which is available to authorized users.

## Background

### Description of the condition

The use of sedative agents in the mechanically ventilated, critically ill population is well established, and the rationale for their use is multifaceted. Sedation is important in reducing patient discomfort and suffering through alleviation of pain and anxiety [1]. Sedation also reduces the autonomic hyperactivity that frequently results when these factors are poorly controlled [2]. Achieving appropriate levels of sedation also facilitates the delivery of care and enhances patient safety by reducing the risks of accidental removal of life-sustaining interventions such as endotracheal tubes or central venous catheters [3].

Despite the benefits of sedation, oversedation is unfavorable, as it minimizes patient interaction with caregivers and family members, delays weaning from the ventilator, may influence risk of delirium, and unnecessarily prolongs intensive care unit (ICU) stay [4]. The optimal sedation regimen should therefore provide adequate sedation with a rapid onset, allow timely recovery after cessation with minimal drug accumulation, lead to minimal adverse effects, and be relatively inexpensive. As a result, guidelines recommend the minimization of intravenous sedation infusions and the use of as small doses of sedatives as possible [5].

Commonly used and widely available ICU sedative agents include propofol and benzodiazepines (such as lorazepam, midazolam, and diazepam) [5–7]. These sedative agents are frequently used in conjunction with analgesic medications, such as opioids [8].

Importantly, the use of these sedative agents is not without adverse effects. Propofol is known to cause hypotension in a significant number of patients [9]. Propofol infusion syndrome, although a rare complication associated with higher doses and a longer duration of treatment, manifests with arrhythmias, rhabdomyolysis, acute kidney injury, myocardial dysfunction and is associated with a very high mortality rate [10]. Benzodiazepines may cause respiratory and cardiovascular depression as well as unintended excessive sedation secondary to drug accumulation in adipose tissue [11]. Benzodiazepines have also been shown to be associated with increased rates of ICU delirium and prolonged mechanical ventilation compared to other non-benzodiazepine sedative agents [12].

Dexmedetomidine is a centrally acting alpha-2 agonist that has sedative properties. Although smaller trials have shown that it may reduce the duration of mechanical ventilation and delirium with a decrease need for alternative sedatives [13–15], it is not universally available mostly related to cost.

### Description of intervention

Clonidine is a centrally acting alpha-2 selective adrenergic agonist similar in action to dexmedetomidine. Traditionally, clonidine has been used to treat attention deficit hyperactivity disorder (ADHD) [16], opioid and alcohol withdrawal [17, 18], hypertension, vasomotor menopausal symptoms, and for neuraxial anesthesia via epidural administration [19, 20]. In the critically ill pediatric population, clonidine is frequently used as a sedative agent, particularly as an adjunctive agent when there is an inadequate response to opioids and benzodiazepines, or to help facilitate weaning from mechanical ventilation [21]. The evidence to support the use of clonidine in the critically ill adult population is less clear. Overall, the current clinical use of clonidine in the critically ill is quite variable [4].

Data supporting the use of clonidine as a sedative agent in the ICU setting remains limited. Known side effects of clonidine include hypotension and rarely bradycardia, as well as rebound tachycardia and hypertension after clonidine withdrawal. Clonidine can be administered via oral, transdermal, or intravenous route. However, only the oral and transdermal formulations are available in North America. Generic versions of clonidine are available, making this intervention extremely inexpensive.

### How the intervention might work

Clonidine is a centrally acting alpha-2 selective adrenergic agonist. It has been postulated that clonidine exerts its sedative effects via stimulation of the pre-synaptic alpha-2 adrenoceptors of the locus coeruleus, decreasing norepinephrine release [22]. Clonidine also has action on the cholinergic, purinergic, and serotonergic pathways, resulting in analgesia [22].

### Why it is important to do this review

The current literature on sedation practices in the critically ill patient population lacks comprehensive summary data on the efficacy of clonidine as a sedative agent. One systematic review, which focused only on pediatric ICU patients, found that adjunctive clonidine use decreased the requirement for other sedative agents, decreased withdrawal symptoms when weaning off benzodiazepines and/or opiates, and was associated with minimal clinically significant adverse effects [21]. Another systematic review specifically examined the role of alpha-2 agonists on sedation in the mechanically ventilated patient population, however focused only on the role of dexmedetomidine [23].

Clonidine is an attractive alternative to other sedating medications given its ease of administration and its improved safety profile. Despite the potential concern regarding hypotension in comparison to dexmedetomidine, clonidine is associated with significant advantages including the availability of an oral formulation and significant cost savings.

### Objectives

We plan to conduct a systematic review of all observational studies, quasi-experimental studies, and RCTs that investigated the use of clonidine as an adjunctive or stand-alone sedative agent in the critically ill population.

## Methods/design

### Types of studies

We plan to include all observational cohort studies that included a control or comparator arm, quasi-experimental studies, and RCTs reporting the use of clonidine as a sedative agent in mechanically ventilated patients. We will exclude case reports, case series, or observational studies that did not include a control/comparator. We will impose no methodological quality restrictions.

### Types of participants

The population of interest includes all patients, including children (under 18 years of age) and adults (18 years of age or older) who require invasive mechanical ventilation (IMV) and sedation. We will exclude studies enrolling exclusively neonates or those that take place exclusively in the neonatal intensive care unit (NICU). If a study includes both IMV and non-invasive ventilation (NIV) patients, we will include the study, but exclude all NIV patients if possible.

### Types of interventions

The intervention of interest is the use of clonidine as a sedative agent for more than 24 h. We will include any mode of delivery of clonidine other than intrathecal. We require the studies to have used clonidine either as an adjunctive or as a stand-alone sedative agent. The comparators or control group will be any standard sedation regimen that does not include clonidine, such as propofol, benzodiazepines, dexmedetomidine, and/or opioids. We will exclude studies using clonidine as a pre-medication for anesthesia or studies in which patients were given clonidine for an alternate indication.

### Types of outcome measures

Our primary outcome is the duration of mechanical ventilation. Secondary outcomes include the following, listed by priority: duration of sedation infusions, dose of sedation used, level of sedation (as assessed by standardized sedation scores such as the Richmond Agitation-Sedation Scale or COMFORT score), incidence of withdrawal from other sedatives, delirium incidence (using the Confusion Assessment Method for the Intensive Care Unit (CAM-ICU), ICU and hospital length of stay, use and duration of NIV, and all-cause ICU and hospital mortality. We will also capture unwanted effects potentially associated with clonidine administration such as clinically significant hypotension or bradycardia (with end-organ dysfunction or requiring intervention), clonidine withdrawal (rebound tachycardia or hypertension), self-extubation, and the accidental removal of central intravenous lines and arterial lines. We will also capture unwanted effects such as clinically significant hypotension or bradycardia (with end-organ dysfunction or requiring intervention), clonidine withdrawal (rebound tachycardia or hypertension), self-extubation, and the accidental removal of central intravenous lines and arterial lines.

### Search methods for identification of studies

We will search the following electronic databases: MEDLINE, EMBASE, ACPJC, CINAHL, and the Cochrane trial registry for eligible articles with no date or language restriction. See appendix for MEDLINE search strategy [Additional file 1]. Keyword search terms include clonidine, sedation, critically ill, mechanical ventilation, delirium, and withdrawal.

### Searching other resources

Two reviewers will independently hand-search the references of review articles and systematic reviews on the same topic for eligible articles. In addition, we will search for unpublished or ongoing trials on the WHO International Clinical Trials Registry (WHO ICTRP), current controlled trials metaregister of controlled trials, clinicaltrials.gov database, and conference proceedings citation index within the last 2 years for the Society of Critical Care Medicine (SCCM), Canadian Critical Care Society, the European Society of Intensive Care Medicine (ESICM), the International Society of Intensive Care and Emergency Medicine (ISICEM), American Thoracic Society (ATS), and the World Federation of Pediatric Intensive and Critical Care Societies.

### Data collection and analysis

After identification of potentially relevant articles, three reviewers (JW, EBC, BR) working in pairs will independently screen all citations and references using specific eligibility criteria. Disagreements will be resolved by discussion and consensus with the help of the third reviewer if needed.

### Data extraction and management

Data extraction will be done independently and in duplicate using pre-designed data abstraction forms (see “Observational abstraction [Additional file 2]” and “RCT abstraction” [Additional file 3]). Abstracted data will include the study title, first author, relevant demographic data, intervention and control, results for outcomes of interest, and information on the methodological quality for each study. A third reviewer will resolve inconsistent data extraction between the two reviewers.

### Assessment of risk of bias in included studies

Two reviewers will independently assess the risk of bias for each included study using the Cochrane Collaboration tool for assessing risk of bias [24] for RCTs and the Ottawa-Newcastle tool for observational studies [25]. Risk of bias assessment will be performed individually per outcome. A third reviewer will be available to resolve any disagreements. For each study, a description for each domain assessed will be included along with comments if necessary and a final judgment. The risk of bias for each study will be categorized as follows: (1) low risk of bias, where bias is not present or if present, unlikely to affect outcomes, (2) high risk of bias, where outcomes are likely to be significantly affected by bias, (3) unclear risk of bias, where there is inadequate reported information to properly assess bias or where it is unclear how much the risk of bias may affect outcomes.

RCTs will be assessed for adequate sequence generation, allocation sequence concealment, blinding, selective outcome reporting, and other bias. Sequence generation will be considered adequate if the study explicitly described an appropriate randomization procedure to generate an unpredictable sequence of allocation, including computerized randomization, use of random number tables, and coin tossing. Concealment of allocation will be considered adequate if specific methods to protect allocation were documented and implemented. Performance bias will be considered low if a study reported participant, caregiver, and/or researcher blinding. Blinding of outcome assessment will be considered adequate if outcome assessors and adjudicators were blinded. Within-study selective reporting of outcomes will be examined by reviewing the a priori study protocol if available. If the study protocol is not available, we will compare the outcomes listed in the “Methods/design” section with those reported in the manuscript.

Observational studies will be assessed for the following: representativeness of the exposed cohort, selection of the non-exposed cohort, ascertainment of exposure, demonstration that the outcome of interest was not present at the start of the study, the comparability of the cohorts on the basis of the design or analysis, outcome assessment methods, and the adequacy of follow-up.

### Measures of treatment effect

When pooling of outcome data is appropriate, RevMan 5.2 software will be used to conduct meta-analyses. RCTs and observational studies will be pooled and analyzed separately. We will use the method of DerSimonian and Liard in random effects model to pool effect sizes for each outcome; study weights will be measured using the inverse variance method. We will present the results as relative risk (RR) with 95 % confidence interval (CI) for dichotomous outcomes and as mean difference (MD) or standardized mean difference (SMD) for continuous outcomes with 95 % CI. We plan to perform random effects analysis for all outcomes of interest. If significant unexplained heterogeneity exists, or if there is an insufficient number of RCTs for meta-analyses, data will be described qualitatively. The number needed to treat (NNT) with 95 % CI will be derived from pooled risk ratios and its 95 % CI utilizing assumed control risk (ACR) for each outcome similar to the approach recommended by the Cochrane collaboration:NNT = 1/[ACR × (1 − RR)] [26].

### Dealing with missing data

Where possible, if missing data is encountered, we will attempt to contact the individual study authors for additional information. If this is not possible, we will analyze the available data and report any potential impact of missing data on the results in the “Discussion” section.

### Assessment of heterogeneity

We will assess for heterogeneity between studies using the chi-squared test for homogeneity, where *p* < 0.01 indicates substantial heterogeneity, and the *I*^2^ statistic. We consider *I*^2^ > 50 % to be significant heterogeneity, which will be further investigated with subgroup analyses to assess clinical and methodological sources of heterogeneity in intervention effect. If there is significant statistical or clinical heterogeneity not explained by subgroup or sensitivity analyses, we will not perform a meta-analysis, and instead, we will describe the data qualitatively.

### Assessment of reporting biases

We will look for potential publication bias using a funnel plot if greater than ten trials are included. For continuous outcomes, the Egger test [24] will be used to detect funnel plot asymmetry. For dichotomous outcomes, the arcsine test will be used. All analyses will be performed using RevMan or R.

### Subgroup analysis and investigation of heterogeneity

Potential and expected clinical sources of heterogeneity include different patient demographics, dosing strategies and delivery of clonidine, non-clonidine sedative regimens, and different methods of capture for certain outcomes such as delirium or level of sedation.

To explore significant heterogeneity, when possible, we will conduct the following subgroup analyses: (1) children (under 18 years of age), hypothesizing that the adult population may benefit more from clonidine, given an increased prevalence of opioid or alcohol withdrawal. (2) Clonidine delivered via enteral vs. parenteral route, hypothesizing that there may be issues with enteral delivery of clonidine in the critically ill due to access (for example, requiring a nasogastric tube), absorption, or tolerance concerns leading to greater benefit with parenteral delivery. (3) Clonidine delivered once or twice per day vs. more frequent dosing, hypothesizing that the more frequent dosing regimen would provide a more predictable and steady concentration of the drug. (4) Early administration (within the first 48 h following intubation) vs. later administration (after the first 48 h following intubation), hypothesizing that earlier use will facilitate weaning or prevent features of withdrawal. (5) Patients who have ongoing opioid or alcohol use leading to a high risk for withdrawal vs. patients without a history of opioid or alcohol use, hypothesizing that withdrawal patients may benefit more from clonidine. (6) Unclear or high risk of bias studies vs. low risk of bias studies, hypothesizing that the studies at high risk of bias will show a larger effect size.

We acknowledge that subgroup analysis may not be possible depending on the number of trials included in the final analysis.

### Sensitivity analysis

A priori sensitivity analysis will be performed excluding studies only reported as abstracts and lacking formal publication. Post hoc sensitivity analysis will be performed where appropriate.

### Assessing the quality of evidence

The Grading of Recommendations Assessment, Development and Evaluation (GRADE) approach will be used to assess the quality of evidence for each outcome [27]. The GRADE system classifies the quality of the aggregate body of evidence as high, moderate, low, or very low. The evidence will be evaluated using the following criteria: (1) study design and rigor of its execution (i.e., individual study risk of bias), (2) the extent to which the evidence could be applied to patients of interest (i.e., directness), (3) the consistency of results, (4) the analysis of the results (i.e., precision), and (5) whether there was a likelihood of publication bias. The following three factors lead to potential upgrading of the quality of evidence if present: (1) a strong or very strong association between an intervention and the observation of interest, (2) a highly statistically significant relationship between dose and effect, and (3) a plausible confounding variable that could explain a reduced effect or could explain an effect if one was not anticipated.

A final overall quality of evidence will be summarized for the intervention taking into consideration both desirable and undesirable outcomes. An evidence profile will be included in the results showing the GRADE assessments and pooled analysis per outcome.

## Discussion

The ideal sedative agent allows for safe and effective sedation practices, thereby potentially reducing the time required to wean from invasive mechanical ventilation and minimizing the use of other sedative-analgesic agents. It should also be associated with minimal adverse effects. Although no perfect agent exists, clonidine may be beneficial compared to available alternatives. This systematic review will summarize the evidence on the efficacy and safety of using clonidine as a sedative agent in critically ill patients. The quality of evidence will be assessed using the GRADE approach to characterize the confidence in the estimate of effect.

## Additional files

Additional file 1:
**An outline of our search strategy.** (XLSX 30 kb) Additional file 2:
**Observational studies data abstraction form.** (DOCX 75 kb) Additional file 3:
**RCT data abstraction form.** (XLSX 26 kb)

## Abbreviations

ACPJC: American College of Physicians Journal Club
ACR: assumed control risk
ADHD: attention deficit hyperactivity disorder
ATS: American Thoracic Society
CAM-ICU: Confusion Assessment Method for the Intensive Care Unit
CI: confidence interval
CINAHL: Cumulative Index to Nursing and Allied Health Literature
ESICM: European Society of Intensive Care Medicine
GRADE: Grading of recommendations assessment development and evaluation
ICU: intensive care unit
IMV: invasive mechanical ventilation
ISICEM: International Society of Intensive Care and Emergency Medicine
MD: mean difference
NICU: neonatal intensive care unit
NIV: non-invasive ventilation
NNT: number needed to treat
RCT: randomized controlled trial
RR: relative risk
SCCM: Society of Critical Care Medicine
SMD: standardized mean difference
WHO ICTRP: World Health Organization International Clinical Trials Registry Platform
