# Supplementary material for: Clonidine for sedation in the critically ill: a systematic review and meta-analysis (protocol)
Source: Syst Rev. 2015 Nov 6;4:154. doi: 10.1186/s13643-015-0139-7 (PMC4635616; doi:10.1186/s13643-015-0139-7)
Supplement: Additional file 1: — An outline of our search strategy. (XLSX 30 kb) [file 13643_2015_139_MOESM1_ESM.docx]

**Search strategy**

Databases

1. MEDLINE
2. EMBASE
3. CINAHL
4. Cochrane registry

|  | Keywords | Adjectives |
| --- | --- | --- |
| Types of studies | - RCTs - Quasi-RCT/experimental studies - Observational prospective cohort studies | - Controlled clinical trial |
| Population | - Mechanically ventilated - ICU/PICU/CCU - Require sedation | - Children, pediatrics, paediatrics, adults, elderly - Artificial respiration, artificial ventilation, intubated. - Intensive care patients, critical care patients - Sedation, sedative agent |
| Interventions | - Clonidine, use intended > 24 hours - Non-clonidine sedative regimen (“standard of care”) | - Catapres, Duraclon, Kapvay, Dixarit, Nexiclon, alpha-2 agonists |
| Outcomes | All clinically relevant | |

MEDLINE MeSH headings

1. Randomized controlled trials as topic
2. Controlled clinical trials as topic
3. Non-randomized controlled trials as Topic
4. Pragmatic clinical trials as topic
5. Observational study as topic
6. Cohort studies
7. Prospective studies
8. Child

*Note MEDLINE MeSH defines “child” as 6-12 yo.

1. Child,preschool

*Note MEDLINE MeSH defines “child, preschool” as 2-6 yo.

1. Pediatrics
2. Adolescent

*Note MEDLINE MeSH defines “adolescent” as person 13-18 yo.

1. Young adult

*Note MEDLINE MeSH defines “young adult” as person 19-24 yo.

1. Adult

*Note MEDLINE MeSH defines “adult” as person 19-44 yo.

1. Middle aged
2. Aged

*Note MEDLINE MeSH defines “aged” as person 65-79.

1. Aged, 80 and over

*Note MEDLINE MeSH defines “aged, 80 and over” as person 65-79.

1. Intensive care
2. Intensive care units
3. Critical care
4. Intensive care units, pediatric
5. Respiration, artificial
6. Positive pressure respiration
7. Intubation, intratracheal
8. Deep sedation
9. Clonidine
10. Adrenergic alpha-2 receptor agonists
11. 1 or 2 or 3 or 4 or 5 or 6 or 7
12. 8 or 9 or 10 or 11 or 12 or 13 or 14 or 15 or 16
13. 17 or 18 or 19 or 20 or 21 or 22 or 23
14. 25 or 26
15. 27 and 28 and 29 and 30 and 24
